# Reproductive Constraints and Severe Pollinator Limitation in the Mexican Endemic Orchid *Govenia capitata*: Implications for Conservation

**DOI:** 10.3390/plants14213377

**Published:** 2025-11-04

**Authors:** Maythe López-Olvera, Gema Galindo-Flores, Ana Laura López-Escamilla, Carlos Lara

**Affiliations:** 1 Maestría en Biotecnología y Manejo de Recursos Naturales, Universidad Autónoma de Tlaxcala (UATX), San Felipe Ixtacuixtla 90120, Tlaxcala, Mexico; loom950825@gmail.com; 2 Centro de Investigación en Ciencias Biológicas, Universidad Autónoma de Tlaxcala (UATX), San Felipe Ixtacuixtla 90120, Tlaxcala, Mexico; gemalilia.galindo.f@uatx.mx; 3 Laboratorio Regional de Biodiversidad y Cultivo de Tejidos Vegetales, Instituto de Biología, Universidad Nacional Autónoma de México, Santa Cruz 90640, Tlaxcala, Mexico; lopezescamilla@ib.unam.mx

**Keywords:** endemic species, inbreeding depression, montane forest, orchid conservation, pollination limitation, seed viability

## Abstract

Understanding the reproductive biology of orchids is essential for evaluating population viability and guiding conservation strategies, as their persistence often depends on complex interactions between ecological, physiological, and environmental factors. *Govenia capitata*, a threatened orchid endemic to the montane forests of central Mexico, had not previously been studied in this regard. We examined flowering phenology, floral longevity, stigmatic receptivity, natural and experimental pollination success, seed viability, and asymbiotic germination in two wild populations. Flowering was synchronous, with inflorescences lasting up to 57 days and individual flowers persisting for an average of 20 days. Stigmatic receptivity was detectable from the first day of anthesis and remained evident for at least eight days. Natural fruit set was very low (16.6%), while assisted self- and cross-pollination reached 100% success, demonstrating self-compatibility despite the inability for autonomous selfing due to floral structure. Seed viability differed significantly among treatments, being lowest in selfed capsules (11%) and highest in cross-pollinated ones (32%), representing a 65% reduction and reflecting severe inbreeding depression that extended to germination performance. In vitro germination success also varied, with the L-arginine medium yielding the highest values (46% for cross-pollinated seeds and 44% for naturally pollinated seeds), though post-germination survival requires optimization for conservation applications. Despite the conspicuous floral display, floral visitation was extremely rare and the pollinator identity remains unknown, with only one potentially effective visitor observed during 144 h of monitoring, and most floral visitors were non-pollinating arthropods such as crab spiders, weevils, hymenopterans, and thrips. Population density varied dramatically (26-fold) between sites separated by less than 1 km, indicating pronounced sensitivity to local environmental conditions. These findings reveal that reproduction in *G. capitata* is constrained by both extrinsic (pollinator limitation) and intrinsic factors (reduced seed viability), which collectively jeopardize long-term population persistence. From a conservation perspective, protecting montane forest remnants and pollinator communities is essential, while the demonstrated potential of asymbiotic germination provides a complementary tool for ex situ propagation and management of this endemic orchid.

## 1. Introduction

Mexico’s mountainous landscapes harbor one of Earth’s most remarkable concentrations of orchid diversity, with 1347 documented species representing 4.8% of global Orchidaceae richness compressed into just 1.3% of the planet’s terrestrial surface [1,2]. This extraordinary floristic wealth reflects the complex geological history and climatic diversity of Mesoamerica, where Tertiary mountain building created numerous isolated ranges that served as evolutionary laboratories for plant diversification [3]. The endemic component of this orchid flora is particularly striking, with 661 species occurring nowhere else on Earth [1].

The conservation status of Mexican orchids presents one of the most pressing botanical conservation challenges in the Neotropics. Current threat assessments indicate that around 40.3% of Mexican endemic orchids are classified under some category of risk according to the Official Mexican Standard [4], while many others likely warrant similar status but remain scientifically understudied. The primary drivers of orchid decline mirror broader patterns of environmental degradation across Mexico: accelerating deforestation has eliminated substantial forest cover since 1950, while agricultural expansion, urbanization, and infrastructure development continue to fragment remaining natural habitats [5]. Climate change adds another layer of complexity, as shifting precipitation patterns and rising temperatures threaten to disrupt the precise environmental conditions that many orchid species require for survival and reproduction [6].

Terrestrial orchids represent the most vulnerable segment of Mexico’s orchid diversity, combining the specialized ecological requirements characteristic of all orchids with additional dependencies that make them particularly sensitive to environmental change [7]. Unlike their epiphytic relatives, terrestrial orchids depend entirely on soil-based mycorrhizal networks for nutrient acquisition and often exhibit extreme specificity in their fungal partnerships [8]. These underground alliances prove remarkably fragile, as soil disturbance, chemical contamination, or changes in forest structure can disrupt mycorrhizal communities and eliminate orchid populations even when suitable habitat appears to persist [9]. Additionally, terrestrial orchids typically require several years to reach reproductive maturity and often exhibit irregular flowering patterns that make population assessment and monitoring particularly challenging [10].

Pine–oak forests represent one of Mexico’s most extensive and ecologically important forest types, covering approximately 16% of the national territory and supporting exceptional levels of endemic flora across elevational gradients from 1200 to 4000 m [11]. These mixed forests create complex habitat mosaics where temperate and subtropical elements intermingle, generating the environmental heterogeneity that has fostered orchid diversification throughout Mexico’s mountain ranges [12]. The transitional nature of pine–oak forests, occupying intermediate positions between tropical and temperate zones, makes them particularly important refugia for endemic species during climate fluctuations [13]. However, pine–oak forests face severe conservation pressures, with significant habitat loss due to agricultural conversion, logging, and urban development during the past century [5]. The remaining forests exist primarily as fragmented patches scattered across Mexico’s mountain ranges, creating a conservation landscape dominated by small, isolated habitat remnants vulnerable to edge effects and local extinction [14]. Climate change compounds these threats, as rising temperatures may force species distributions upslope while simultaneously reducing the total area of suitable habitat at higher elevations [15].

These fragmentation patterns create particularly complex challenges for the specialized organisms that depend on pine–oak forest ecosystems. The interaction between habitat fragmentation and reproductive biology creates particularly acute conservation challenges for pine–oak forest orchids. Many species depend on animal pollinators that may themselves be affected by habitat changes, creating the potential for pollination networks to collapse even when both plants and pollinators persist in modified landscapes [16]. The complex life cycles of orchids, involving dust-like seeds that require specific mycorrhizal partners for germination, mean that recruitment failure can occur through disruption of any component of these intricate ecological relationships [17]. Understanding these reproductive dependencies becomes essential for developing effective conservation strategies that address the full spectrum of threats facing endemic orchid populations [18].

The genus *Govenia* Lindl. exemplifies both the conservation challenges and the knowledge gaps that characterize Mexican endemic orchids. Phylogenetic analyses suggest that this group underwent diversification across Mexico’s mountain ranges, with multiple lineages evolving in isolation [19]. This evolutionary history has produced a remarkable assemblage of Mexican species, including endemics that represent evolutionary legacies of past climate changes. Contemporary species exhibit pronounced ecological specialization, with most taxa occupying narrow elevational ranges in specific forest types [19,20], suggesting limited ability to track suitable habitat under future climate scenarios. Despite their evolutionary significance and apparent conservation vulnerability, fundamental biological knowledge remains lacking for most *Govenia* species. Previous studies have provided tantalizing glimpses into the reproductive ecology of the genus, suggesting that species employ deceptive pollination strategies in which flowers attract pollinators without providing actual rewards to visitors [21]. This reproductive strategy, while enabling resource conservation and potentially reducing competition for pollinators, creates inherent vulnerability to pollination failure if appropriate pollinators become scarce or if environmental changes disrupt pollinator–plant interactions.

**Figure 1:**
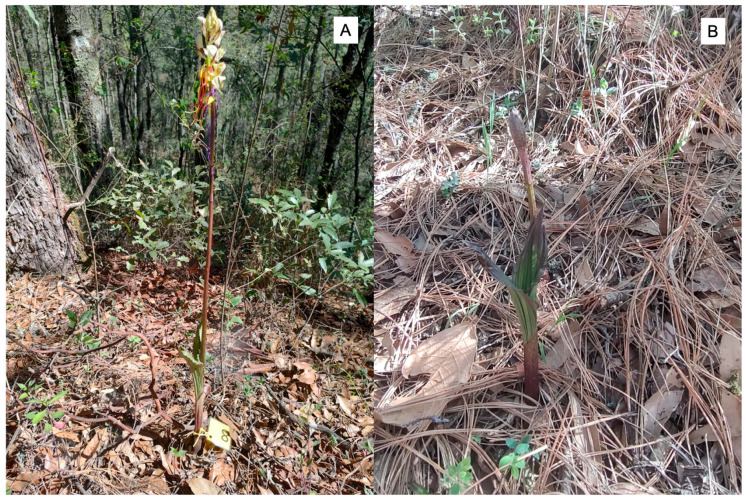
*Govenia capitata* in its natural pine–oak forest habitat in central Mexico. (**A**) Mature flowering individual displaying full inflorescence development with multiple flowers in anthesis, illustrating the species’ growth habit within the mixed *Pinus montezumae*-*Quercus crassipes* forest ecosystem at approximately 2890 m elevation in Tlaxcala, Mexico. (**B**) Early flowering stage showing characteristic pleated leaves with prominent parallel venation and emerging inflorescence, surrounded by typical understory conditions with dense leaf litter accumulation. Both images demonstrate the partially shaded microhabitat preferences and terrestrial growth form characteristic of the species.

*Govenia capitata* Lindl. exemplifies the significant knowledge gaps that characterize many species within this genus. This orchid is a terrestrial species reaching 40–80 cm in height, characterized by distinctive pleated leaves and dense terminal racemes bearing 6–18 white to cream-colored flowers. The species exhibits the typical geophytic growth form of the genus, with underground pseudobulbous corms that enable survival through seasonal dormancy. Inflorescences emerge during early summer, displaying flowers with prominent labella marked by distinctive purple–brown spotting patterns (Figure 1). The species has maintained a limited presence in botanical literature, appearing in floristic accounts and herbarium records but lacking comprehensive biological studies [20]. Historical collections document its presence across central Mexico, yet field encounters remain infrequent, suggesting either naturally low population densities or ecological characteristics that make detection challenging [22]. The ecological requirements of *G. capitata* appear highly specific, with all verified records coming from pine–oak forest between 2000 and 3000 m elevation where cool, humid conditions characteristic of montane environments create suitable microhabitat [20]. The species exhibits the terrestrial habit typical of its genus, with underground storage organs (corms) that enable survival through dry seasons when aerial parts die back. This geophytic strategy provides resilience against seasonal environmental variation but also makes population monitoring challenging, as plants may remain underground for extended periods and only become visible during brief reproductive episodes [23]. Recent field observations have documented the species at high elevations, such as encounters at approximately 2885 m in oak forests of Querétaro [22], expanding our understanding of *G. capitata* distribution and suggesting the potential for continued discoveries in Mexico’s mountainous regions. These discoveries highlight both the potential for continued biological exploration in Mexico’s mountains and the urgent need for systematic study of documented populations before they potentially disappear due to ongoing environmental changes.

Despite these recent discoveries and the species’ apparent conservation importance, critical aspects of its biology remain unexplored. The reproductive biology of *G. capitata* has remained completely unstudied despite its conservation significance and the documented threats facing its pine–oak forest habitat. Understanding how this species achieves reproduction becomes critical for assessing its vulnerability to environmental change and developing appropriate conservation strategies. Key questions include whether the species depends on specific pollinators that may themselves be threatened, whether small population sizes create reproductive limitations through pollen limitation or genetic bottlenecks, and whether ex situ propagation techniques could provide demographic support for declining wild populations. Contemporary orchid conservation has increasingly embraced integrated approaches that combine habitat protection with ex situ propagation techniques, recognizing that many threatened species require active intervention to prevent extinction [8,24]. Asymbiotic seed germination protocols have revolutionized orchid conservation by enabling rapid propagation of threatened genotypes without the need to isolate and culture appropriate mycorrhizal fungi [23]. However, these techniques require species-specific optimization, as germination requirements vary dramatically across orchid taxa depending on seed morphology, dormancy mechanisms, and nutritional requirements [25].

The development of effective propagation protocols for *G. capitata* could provide essential tools for population recovery efforts while also serving as a model for conservation of other threatened *Govenia* species [26]. Success in this endeavor requires detailed understanding of seed biology, including factors affecting viability, optimal germination conditions, and post-germination survival requirements [27]. Such knowledge proves particularly valuable for species occurring in highly fragmented habitats where natural recruitment may be compromised by disrupted ecological relationships [28]. Here, we present the first comprehensive investigation of *G. capitata* reproductive biology, integrating field experiments, observational ecology, and laboratory propagation trials. Our objectives were to: (1) characterize flowering phenology and reproductive timing; (2) determine breeding system compatibility and pollination requirements; (3) identify floral visitors and assess pollination effectiveness; (4) quantify population density; (5) evaluate seed quality across pollination treatments; and (6) develop asymbiotic germination protocols for conservation applications. The knowledge generated through this study addresses critical information gaps that have limited conservation planning for this species while also providing insights relevant to the broader challenge of conserving Mexico’s threatened orchid diversity.

## 2. Results

### 2.1. Flowering Phenology and Synchrony

**Figure 2:**
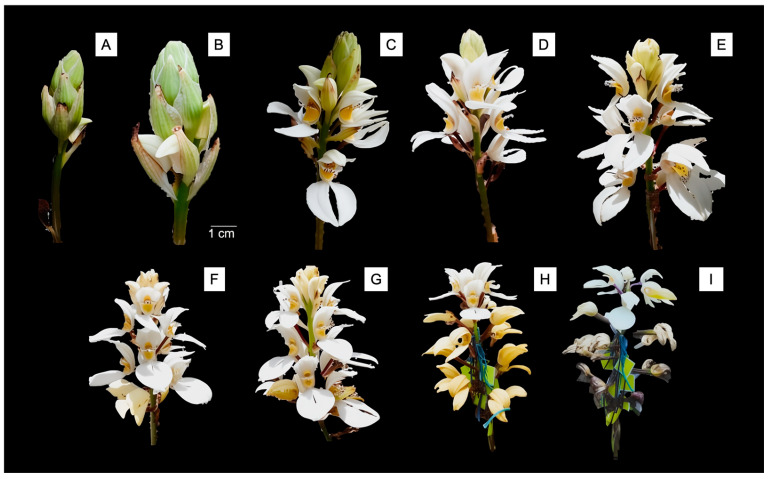
Sequential development and senescence of a *Govenia capitata* inflorescence. The series illustrates the progression from (**A**–**C**) bud initiation, (**D**–**F**) anthesis phases, (**G**) peak flowering, and (**H**,**I**) floral senescence stages, highlighting the acropetal flowering sequence characteristic of the species. The colored plastic markers with threads visible in panels (**H**,**I**) were used to tag individual flowers for developmental tracking throughout the study period.

**Figure 3:**
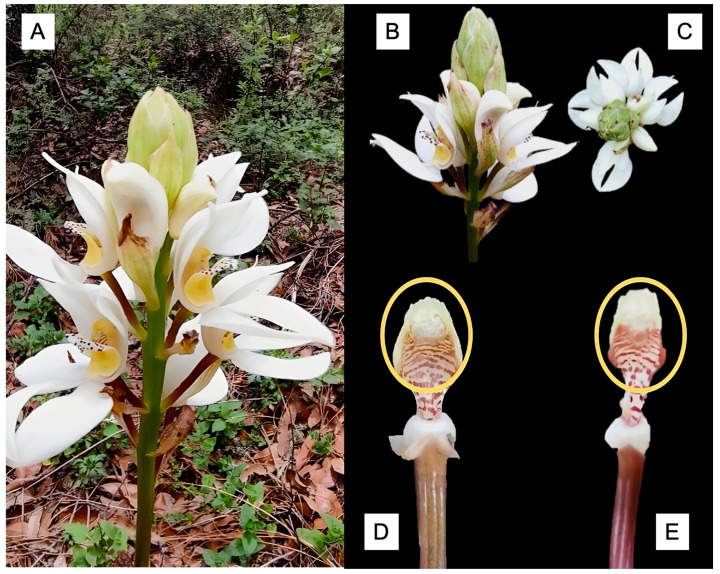
Floral development and stigmatic receptivity in *Govenia capitata*. (**A**) Complete plant showing inflorescence architecture. (**B**) Inflorescences with flowers at different developmental stages, illustrating the acropetal sequence of anthesis. (**C**) Stigmatic bubbling as an indicator of receptivity, observed in (**D**) a day-1 flower and (**E**) a day-8 flower; the receptive area of the stigma is highlighted with a yellow circle.

*G. capitata* exhibited highly synchronous flowering concentrated during early summer months. Circular statistical analysis revealed significant seasonality (Rayleigh test: Z = 235.781, *p* < 0.001) with high synchrony (vector length r = 0.956, indicating that most flowering events clustered tightly around the mean date) and mean flowering date of June 5th (μ = 216.815°). Individual flower longevity averaged 20.26 ± 4.34 days (range: 15–25 days, n = 195), while complete inflorescences remained active for 44–57 days, illustrating the sequential maturation and senescence across racemes (Figure 2). Inflorescences developed acropetally with 6–18 flowers per raceme (mean = 11.7 ± 3.2), providing extended opportunities for pollination throughout the reproductive season. Peroxidase activity confirmed stigmatic receptivity from day 1 through at least day 8 post-anthesis, indicating extended temporal windows for successful pollination throughout individual flower lifespans (Figure 3).

### 2.2. Breeding System and Reproductive Limitation

**Figure 4:**
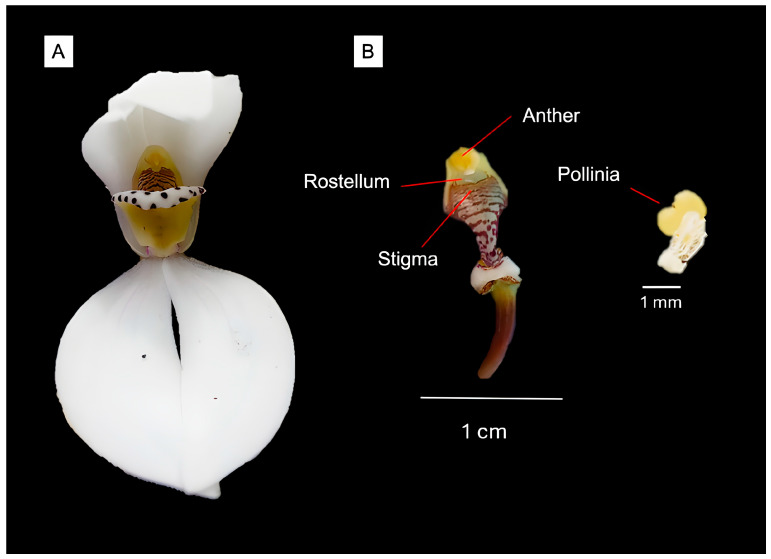
Floral morphology and reproductive structures of *Govenia capitata*. (**A**) Frontal view of a single flower showing the overall structure and the position of the column (gynostemium). (**B**) Close-up of the gynostemium illustrating the anther, rostellum, and stigma. The rostellum acts as a physical barrier separating the pollinia from the receptive stigma, preventing spontaneous self-pollination. The pair of compact pollinia is shown to the right. Scale bars = 1 cm (**B**) and 1 mm (pollinia).

Controlled pollination experiments revealed dramatic variation in reproductive success among treatments, with an 83.4% gap between potential and realized reproduction (Table 1; χ^2^ = 41.287, df = 3, *p* < 0.001). Both cross-pollination and assisted self-pollination achieved complete reproductive success (100% fruit set, 12/12 flowers each), demonstrating full self-compatibility without early-acting inbreeding depression. In contrast, natural pollination yielded only 16.6% fruit set (2/12 flowers), while autonomous selfing failed entirely (0/12 flowers), confirming that floral morphology prevents spontaneous pollen transfer and establishes absolute dependence on pollinator activity for reproductive success (Figure 4).

### 2.3. Capsule Development and Maternal Investment

**Figure 5:**
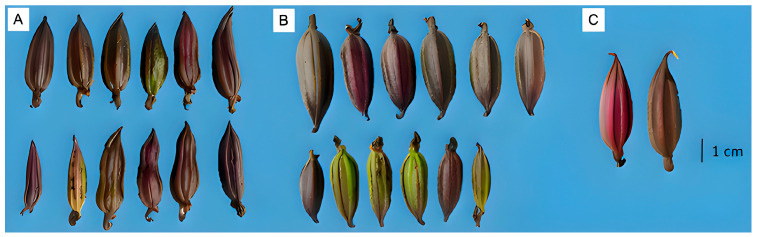
Capsules of *Govenia capitata* obtained under different pollination treatments: (**A**) autonomous self-pollination, (**B**) cross-pollination, and (**C**) open pollination. Scale bars = 1 cm.

Cross-pollinated capsules were significantly larger and heavier than self-pollinated capsules, demonstrating differential patterns of resource allocation among pollination treatments (Table 1, Figure 5). Cross-pollination produced capsules averaging 3.74 ± 0.63 g compared to 2.63 ± 0.44 g for self-pollination (F_2,21_ = 5.444, *p* < 0.01), with corresponding differences in capsule width (1.38 ± 0.27 cm vs. 1.12 ± 0.24 cm; F_2,21_ = 6.334, *p* < 0.006). Capsule length showed no significant treatment effects (F_2,21_ = 0.936, *p* > 0.05). Natural pollination produced intermediate values for both mass (1.93 ± 0.43 g) and width (1.35 ± 0.07 cm).

### 2.4. Visitor Community and Pollination Ecology

**Figure 6:**
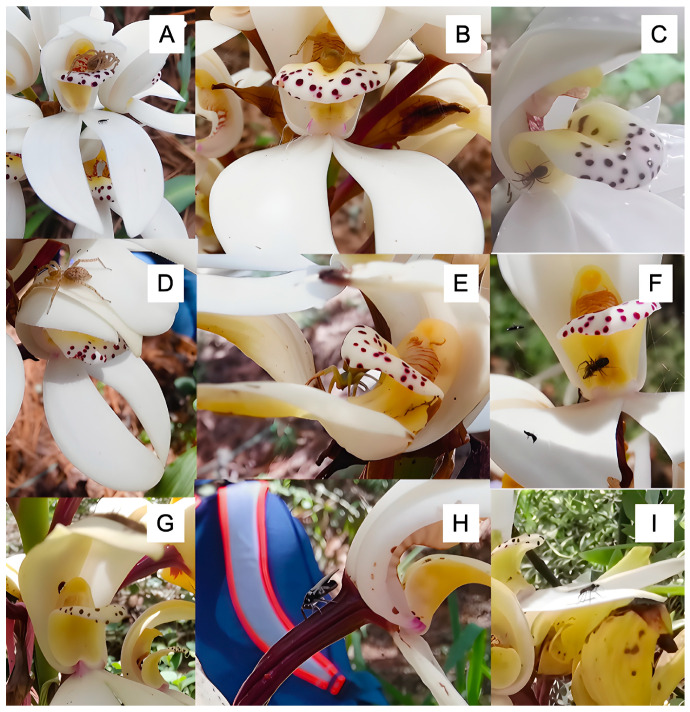
Floral visitors of *Govenia capitata* recorded during the study. (**A**–**F**) Crab spiders (Thomisidae) occupying the labellum. (**G**) A weevil (Curculionidae). (**H**) A hymenopteran visitor (Vespidae). (**I**) Thrips (Thysanoptera) on the perianth.

Despite intensive observation totaling 144 h, floral visitation was remarkably sparse, with only 33 individual arthropod visits recorded across all observation periods (Table 2). The visitor community was dominated by non-mutualistic taxa showing no apparent pollination behavior (Figure 6). Crab spiders (Thomisidae) comprised the most frequent visitors (60.6% of observations, 20 individuals) but engaged exclusively in predatory behavior, using flowers as hunting platforms to ambush small arthropods. Most significantly, only one potentially effective visitor was observed during the entire study—a single hymenopteran individual (Vespidae) that displayed apparent nectar-seeking behavior and made contact with reproductive structures. However, we could not confirm pollinia removal or deposition by this individual. Given the small size of the observed Vespidae visitor compared to the previously reported larger visitors (*Euglossine* bees and *Apis mellifera*), size compatibility with pollinia remains questionable, and the actual pollinator(s) responsible for the 16.6% natural fruit set remain unidentified. This represents a putative pollinator visitation rate of 0.007 visits per hour, compared to a total arthropod visitation rate of 0.23 visits per hour.

### 2.5. Population Density Variation

Population surveys revealed striking density differences between study sites, with Granja Salma supporting 109.69 plants km^−2^ (91 individuals detected) compared to Las Minas with 4.23 plants km^−2^ (8 individuals detected). This 26-fold density variation occurred within sites separated by less than 1 km and sharing broadly similar pine–oak forest structure, suggesting that subtle differences in microhabitat conditions (soil characteristics, mycorrhizal fungal communities, microclimate, or disturbance history) may strongly influence local population establishment. However, sampling of only two sites limits our ability to generalize about habitat requirements across the species’ range.

### 2.6. Seed Quality and Inbreeding Effects

Seed viability assessment revealed significant treatment effects, with pronounced differences between outcrossed and selfed offspring (χ^2^ = 101.78, df = 2, *p* < 0.001). Cross-pollinated seeds showed highest viability (32.54 ± 9.4%), closely followed by naturally pollinated seeds (30.18 ± 8.1%), while self-pollinated seeds exhibited dramatically reduced viability (11.30 ± 4.2%). This represents a 65% reduction in viability for selfed compared to outcrossed offspring. The intermediate viability of naturally pollinated seeds (30.18%) compared to cross-pollinated (32.54%) and self-pollinated seeds (11.30%) may reflect predominantly geitonogamous pollen transfer within the large inflorescences (6–18 flowers per raceme) rather than true xenogamy, given the extremely low pollinator visitation rates observed. Embryo presence rates paralleled viability patterns: 89.5% for cross-pollinated seeds, 86.7% for naturally pollinated seeds, and 69.6% for self-pollinated seeds.

### 2.7. Asymbiotic Germination and Propagation Potential

Laboratory germination trials achieved substantial success, with germination rates varying significantly among medium treatments and seed sources (F_2,18_ = 18.9, *p* < 0.001; Table 3). L-arginine supplementation produced optimal results across all seed types, achieving 46.16% germination for cross-pollinated seeds compared to 22.1% for basal medium—a more than two-fold improvement. Self-pollinated seeds consistently showed reduced germination across all medium treatments (22.68% maximum vs. 46.16% for cross-pollinated seeds), reinforcing the fitness consequences of inbreeding documented through viability assessments. Germination initiated 41 days post-sowing across all treatments, with peak activity occurring during weeks 6–12. However, post-germination survival presented a critical limitation, with progressive chlorosis and tissue necrosis affecting 70–85% of developing seedlings after 85–170 days in culture, limiting current propagation potential for conservation applications.

## 3. Discussion

Understanding the reproductive constraints that limit population viability in endemic orchids requires integration of breeding system analysis, pollination ecology, and population demography. Our comprehensive approach to *Govenia capitata* reveals a species characterized by reproductive flexibility coupled with severe ecological limitations. The breeding system exhibits complete self-compatibility without early-acting inbreeding depression, yet natural fruit set remains dramatically reduced relative to experimental treatments. This reproductive shortfall appears to stem from pollinator limitation rather than intrinsic physiological constraints, as evidenced by the striking disparity between natural (16.6%) and experimentally assisted reproduction (100%). The pronounced inbreeding depression observed in seed viability (65% reduction in selfed offspring) indicates that while self-compatibility provides reproductive assurance, outcrossing remains strongly favored. These findings suggest that the species maintains a genetic load that only becomes evident under selfing, a pattern consistent with other orchids where long-term outcrossing predominates but reproductive assurance mechanisms persist for periods of pollinator scarcity [29,30]. Population structure analysis reveals extreme habitat selectivity, with density differences exceeding 26-fold between sites separated by less than 1 km. These patterns suggest that *G. capitata* represents a specialized endemic facing multiple interacting constraints that collectively limit population growth and persistence.

The complete success of both cross-pollination and assisted self-pollination treatments (100% fruit set for both) demonstrates that reproductive failure in natural populations is not caused by physiological incompatibilities or developmental constraints within the breeding system itself. The contrast between experimental success and natural failure (16.6% fruit set) directly implicates pollinator limitation as the proximate mechanism constraining reproduction in wild populations [31,32].

### 3.1. Breeding System and Reproductive Strategy

The self-compatibility demonstrated through our controlled experiments places *G. capitata* among the majority of orchid species that possess the ability to reproduce through both selfing and outcrossing [32]. However, comparisons within the genus *Govenia* are severely constrained by the paucity of reproductive biology studies, with *G. utriculata* representing the only other species for which detailed pollination data exist. In that species, Pansarin (2008) [21] described obligate dependence on syrphid flies through a pollen-deceptive system that achieved 18.2% natural fruit set, remarkably similar to our finding of 16.6% in *G. capitata*. This convergence suggests that low natural reproductive success may be characteristic of the genus, though the limited data prevent broader generalizations. The complete failure of autonomous selfing (0% fruit set) combined with successful assisted self-pollination (100% fruit set) indicates that while the species possesses the genetic and physiological mechanisms for self-fertilization, mechanical barriers or pollinator activity remain necessary for pollen transfer to occur, a pattern consistent with other deceptive orchids that require vector-mediated pollen transfer [33].

The extended stigmatic receptivity documented in our study (at least eight days post-anthesis) provides multiple opportunities for successful pollination throughout the flower’s lifespan. This temporal flexibility is consistent with adaptations to unpredictable pollination environments [34]. The magnitude of inbreeding depression observed in seed viability (32.54% for outcrossed vs. 11.30% for selfed seeds) demonstrates substantial fitness costs associated with self-fertilization. Moreover, this inbreeding depression extended to germination performance, reinforcing that fitness reductions occur across multiple life stages and not only at the seed viability level. This pattern highlights the evolutionary advantage of outcrossing whenever effective pollinators are available. This 65% reduction in viability contrasts with the lack of seed quality data in the only other reproductive study of the genus, where Pansarin (2008) [21] did not assess inbreeding effects in *G. utriculata*. The degree of inbreeding depression we observed falls within the upper range reported for orchid species generally [32], suggesting that *G. capitata* populations have historically maintained sufficient size and outcrossing rates to accumulate genetic load. This pattern indicates that while *G. capitata* possesses the genetic mechanisms for self-compatibility, the species remains obligately allogamous due to floral morphology that prevents autonomous selfing, making reproduction entirely dependent on pollinator activity.

### 3.2. Pollinator Scarcity and Visitor Community

Our systematic observations documented extremely low arthropod visitation rates (33 individuals during 144 h) with a visitor community dominated by non-mutualistic taxa. While we could not definitively identify the pollinator(s) responsible for natural reproduction, the experimental evidence strongly indicates pollinator limitation as the primary constraint. This pattern of low visitation frequency is consistent with studies of other rare orchids experiencing reproductive limitations [35]. Crab spiders (Thomisidae) comprised 60.6% of all floral visitors but engaged exclusively in predatory behavior without contributing to pollination services, a phenomenon documented in other plant species where flower-dwelling predators may deter legitimate pollinators [36]. The effective pollinator community thus appears functionally absent, and the disconnect between conspicuous floral display and actual pollen transfer suggests a breakdown of expected plant–pollinator interactions [32,37].

The documentation of only one potentially effective pollinator during the entire observation period provides direct evidence for the pollinator scarcity that accounts for low natural fruit set. Previous observations noted visits by bees of the genus Euglossine and honey bees (*Apis mellifera*) to *G. capitata*, suggesting these as potential pollinators [38], yet our intensive field observations during peak flowering revealed minimal effective pollination activity, indicating possible temporal or spatial mismatches in pollinator availability. While direct comparisons with other *Govenia* species are limited by the scarcity of pollination studies in this genus, the specialized syrphid fly pollination system documented in *G. utriculata* by Pansarin (2008) [21] suggests that *Govenia* species may generally depend on specific pollinator groups that are vulnerable to environmental disruption. The apparent breakdown of pollinator relationships in *G. capitata* may reflect broader changes in montane forest arthropod communities or temporal mismatches between flowering and pollinator activity cycles, though the absence of comparative data for other *Govenia* species limits our ability to determine whether such pollination failure represents a species-specific phenomenon or a broader pattern within the genus. Such extreme pollinator limitation has been documented in other orchid species, particularly those with specialized pollination systems [33].

The behavioral observations confirm that most floral visitors do not contact reproductive structures in ways that would facilitate pollen transfer. This distinction between visitors and effective pollinators is critical for understanding reproductive success in plant populations [39].

### 3.3. Population Density and Habitat Requirements

The 26-fold difference in population density between our study sites (4.23 vs. 109.69 plants km^−2^) reveals substantial local variation in population size within broadly similar pine–oak forest. Such variation in local densities is characteristic of orchid species with specialized ecological requirements and has been documented in other terrestrial orchids [8,9].

However, our density estimates derive from only two geographically proximate sites within a single municipality, limiting our ability to characterize habitat requirements at broader spatial scales. While the dramatic density difference suggests environmental sensitivity, the specific factors distinguishing high-density from low-density sites remain speculative and could include edaphic variables (soil pH, organic matter content, mycorrhizal fungal diversity), microclimatic differences (moisture availability, temperature variation), forest stand characteristics (canopy structure, understory composition), disturbance history (logging, fire), or stochastic colonization patterns. Extrapolating to “extreme habitat selectivity” at the species level would be premature without surveys across the broader geographic and elevational range of *G. capitata*. This pattern aligns with previous descriptions by Padrón Hernández (2005) [23], who noted the restriction to pine–oak forests at 2000–3000 m with abundant leaf litter, though systematic habitat characterization comparing multiple high-density and low-density sites is essential to identify the specific environmental variables that determine population establishment and persistence.

The existence of the high-density population at Granja Salma demonstrates that suitable microhabitat can support substantial numbers of reproductive individuals, indicating that habitat quality differences, rather than broader environmental constraints, primarily determine local population sizes [24,40].

### 3.4. Seed Quality and Maternal Investment

Cross-pollinated capsules were significantly larger and heavier than self-pollinated capsules (3.74 ± 0.63 g vs. 2.63 ± 0.44 g), indicating differential maternal resource allocation between outcrossed and selfed offspring. This pattern of increased maternal investment in outcrossed offspring has been documented in other plant species and reflects the higher fitness value of genetically diverse progeny [41,42].

The seed viability differences between pollination treatments (32.54% for cross-pollination vs. 11.30% for self-pollination) demonstrate measurable fitness consequences of inbreeding. These results provide direct evidence that genetic diversity benefits offspring survival in *G. capitata*, consistent with patterns documented across diverse plant taxa [30,43].

The similar viability rates between naturally pollinated seeds (30.18%) and cross-pollinated seeds (32.54%) indicate that natural pollination events, when they occur, predominantly result in outcrossing rather than self-fertilization.

### 3.5. Ex Situ Propagation Development

Our asymbiotic germination trials successfully achieved substantial germination rates, with L-arginine supplementation producing optimal results (46.16% germination). This result aligns with evidence that amino acid supplementation enhances orchid germination [8,24,44,45] and emphasizes its practical conservation relevance. Importantly, the superior germination of cross-pollinated seeds compared to selfed ones also highlights the ecological costs of inbreeding, underscoring the necessity of maintaining genetic diversity in both in situ and ex situ conservation strategies. This enhancement represents a significant improvement over basal medium conditions (22.1%) and compares favorably with germination rates reported for other terrestrial orchids using asymbiotic protocols [24,46]. These results complement the earlier asymbiotic germination success achieved by Padrón Hernández (2005) [23] using Knudson “C” medium supplemented with potato extract, though that study documented an exceptionally long development period of 690 days from seed to complete plant, representing one of the longest germination periods reported for orchid species. The beneficial effect of L-arginine supplementation adds to growing evidence that amino acid metabolism plays an important role in orchid seed germination [47,48].

The consistent superiority of cross-pollinated seeds in germination trials reinforces the fitness advantages of outcrossing documented through viability assessments. Even under controlled laboratory conditions, selfed offspring showed reduced performance across all medium treatments, consistent with the expression of inbreeding depression throughout early life stages [29,49].

The high post-germination mortality observed (70–85% of germinated seedlings) identifies a critical limitation in current propagation protocols that requires resolution before operational conservation breeding becomes feasible. This mortality pattern is common in orchid propagation efforts and typically reflects the absence of mycorrhizal partnerships essential for continued development [50,51].

### 3.6. Conservation Implications

The reproductive biology documented in this study identifies specific mechanisms limiting *G. capitata* population growth and provides guidance for targeted conservation interventions. The complete self-compatibility combined with severe pollination limitation suggests that assisted reproduction could provide immediate demographic benefits to declining populations, an approach that has proven successful for other threatened orchids [8,26]. The potential for ex situ conservation is particularly promising given the remarkable capacity for somatic embryogenesis documented by Padrón Hernández (2005) [23], who demonstrated the generation of over 1080 somatic embryos from callus tissue without hormone supplementation. This “enormous potential for multiplication” suggests that *G. capitata* could be propagated at scale for restoration purposes without depleting wild seed resources, offering a complementary approach to the pollination augmentation strategies indicated by our field results.

The successful development of germination protocols offers tools for ex situ conservation, though improvements in post-germination survival are necessary for large-scale application. The genetic consequences of inbreeding depression emphasize the importance of maintaining genetic diversity in any conservation breeding programs, consistent with established principles of conservation genetics [52,53].

The extreme habitat selectivity documented through population surveys indicates that conservation efforts should prioritize protection of sites supporting high-density populations while investigating the environmental factors that distinguish optimal from suboptimal habitat [9,54].

### 3.7. Research Priorities and Future Directions

Our findings identify several critical research needs for effective *G. capitata* conservation. Pollinator identification studies are essential for understanding the ecological requirements of the species’ mutualistic partners and assessing their conservation status [28,33]. Such research should employ targeted sampling during peak flowering periods and examine pollinator community composition across multiple sites and years.

Habitat characterization studies comparing high-density and low-density sites could identify specific environmental factors that determine site suitability. Such research should examine soil chemistry, mycorrhizal fungal communities, forest structure variables, and microclimate conditions that may influence population establishment and growth [9,55].

Improved propagation protocols addressing post-germination mortality are necessary for operational conservation breeding. This research should focus on mycorrhizal inoculation techniques and nutritional requirements during the transition from laboratory culture to independent growth [56,57]. Population monitoring programs are essential for tracking demographic trends and assessing the effectiveness of conservation interventions. Such monitoring should integrate population counts with reproductive success measurements to provide early detection of population changes [58,59].

### 3.8. Study Limitations

Several limitations of our study should be acknowledged when interpreting these results. Our observations were restricted to a single flowering season (2023) and two geographically proximate sites, which may not capture the full range of temporal and spatial variation in reproductive success and population dynamics across the species’ range. The systematic pollinator observations, while intensive (144 h), were concentrated during daylight hours and may have missed crepuscular or nocturnal pollination activity. Additionally, our visitor identification was limited by the challenges of taxonomic determination in the field, particularly for small arthropods that could not be collected.

The sample sizes for controlled pollination experiments (n = 12 per treatment) were adequate for detecting large treatment effects but may have limited power to detect subtle differences in reproductive success. The asymbiotic germination protocols, while successful in achieving initial germination, require further optimization to address post-germination mortality before they can be applied operationally for conservation purposes.

Finally, our population surveys employed visual detection methods that may have underestimated true population sizes if non-reproductive individuals or those in vegetative stages were present but not detected. Despite these limitations, our findings provide the first comprehensive assessment of *G. capitata* reproductive biology and establish a baseline for future comparative studies.

## 4. Materials and Methods

### 4.1. Study System and Sites

**Figure 7:**
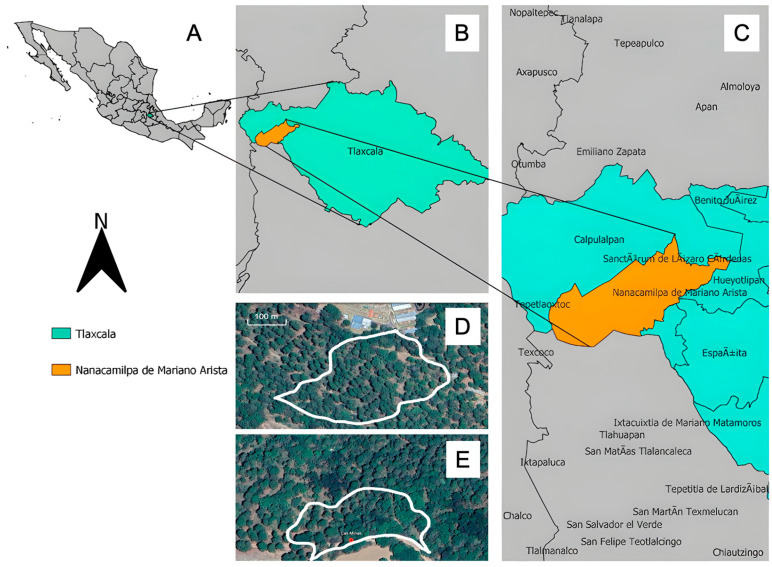
(**A**) Map of Mexico with Tlaxcala state highlighted in green. (**B**) Tlaxcala state showing the location of Nanacamilpa municipality. (**C**) Detailed view of Nanacamilpa municipality in orange. Aerial views of (**D**) Granja Salma and (**E**) Las Minas study sites. White lines indicate approximate boundaries of the orchid populations.

We conducted fieldwork during the complete 2023 flowering season (June–August) in two pine–oak forest sites within Nanacamilpa de Mariano Arista municipality, Tlaxcala, Mexico. “Granja Salma” (19°28′20.76″ N, 98°35′13.31″ W, 2892 m. a.s.l.) and “Las Minas” (19°28′34.15″ N, 98°35′17.82″ W, 2898 m. a.s.l.) represent well-preserved forest remnants characteristic of *G. capitata* habitat (Figure 7). Both sites feature temperate subhumid climate with concentrated summer rainfall (700–1000 mm annually), mean annual temperature of 12–14 °C, and frequent fog during the growing season. Vegetation consists of mixed *Pinus montezumae*-*Quercus crassipes* forest with diverse understory including abundant ferns and deep organic soil layers typical of montane forest environments. *G. capitata* populations occur in partially shaded areas with high leaf litter accumulation and consistent soil moisture.

### 4.2. Flowering Phenology and Reproductive Timing

We monitored 11 reproductive individuals encompassing 195 individual flowers throughout the flowering season. Each flower received unique identification tags at bud stage, enabling precise tracking of developmental timing. Daily observations recorded were as follows: (1) anthesis initiation date; (2) flower condition and appearance; (3) senescence or fruit development. We documented inflorescence architecture, flower number per raceme, and development patterns.

Phenological analysis employed circular statistics using Oriana 4.0 software [60]. Each observation date was converted to angular values (α = 360° × Julian day ÷ 365), enabling calculation of mean flowering date (μ), concentration parameter (r), and seasonal uniformity assessment via Rayleigh tests. Flowering duration was classified following [61]: brief (<1 month), intermediate (1–5 months), or extended (>5 months).

### 4.3. Stigmatic Receptivity Assessment

We tested stigmatic receptivity using enzymatic peroxidase detection [62] at 1 and 8 days post-anthesis on fresh flowers (n = 2 per time point). Stigmatic surfaces were treated with 3% hydrogen peroxide solution, with vigorous bubble formation indicating active peroxidase enzyme and receptive condition.

### 4.4. Controlled Pollination Experiments

Breeding system analysis employed four replicated treatments with 12 flowers each: (1) Natural pollination (control)—flowers marked but unmanipulated to assess natural reproductive success; (2) Autonomous selfing—flowers bagged with fine mesh throughout development to exclude all potential vectors; (3) Assisted self-pollination—flowers emasculated and hand-pollinated using pollen from the same inflorescence after 24 h isolation; (4) Cross-pollination—flowers emasculated and hand-pollinated using pollen from different plants separated by >10 m.

All experimental flowers were individually bagged before and after treatments using fine mesh that permitted air circulation while excluding arthropod visitors. Pollinia were transferred directly to stigmatic surfaces using flame-sterilized forceps. Fruit development was monitored weekly for 16 weeks, with successful fruit set defined as persistent capsule development with green coloration maintained >8 weeks post-treatment. Treatment effects were analyzed using chi-square goodness-of-fit tests.

### 4.5. Floral Visitor Documentation

Systematic visitor observations totaled 144 h across the flowering season, employing randomized 10 min focal watches on marked inflorescences between 10:00 and 16:00 h. We recorded all arthropod visitors contacting flowers, documenting: species identity, visit duration, flowers contacted per visit, behavioral sequences, and apparent foraging objectives. Visitors were photographed in situ and collected when possible for taxonomic verification.

Video monitoring using GoPro cameras supplemented direct observations during peak activity periods. All specimens were preserved in 70% ethanol and identified using regional keys. We attempted to assess pollination effectiveness by examining collected individuals for pollen loads and observing for pollinia removal/deposition during controlled observations, though no definitive pollinator identification was achieved.

### 4.6. Population Structure Assessment

Population surveys employed distance sampling methodology with parallel 100 m transects spaced 50 m apart across both study sites. All detected individuals were mapped using GPS coordinates with perpendicular distances from transect centerlines recorded. Three replicate surveys during early, peak, and late flowering periods ensured detection of all reproductive individuals. Density estimates were calculated following Whitesides et al. (1988) [63] using D = N/2LW, where N = number of individuals observed, L = total transect length, and W = average perpendicular distance.

### 4.7. Seed Biology and Capsule Characteristics

Mature capsules were collected 16 weeks post-pollination from all successful pollination treatments (n = 26 total). Fresh capsules were immediately measured for length, maximum width, and mass using digital calipers (±0.01 mm) and analytical balance (±0.001 g). Morphological differences among treatments were analyzed using one-way ANOVA with Tukey post hoc comparisons.

### 4.8. Seed Viability Assessment

Seed viability was determined using 2,3,5-triphenyl tetrazolium chloride (TTC) staining following established protocols [64,65]. Seeds were pre-conditioned in 10% sucrose solution for 24 h at room temperature, then incubated in 1% TTC solution (pH 6.5) for 24 h in darkness. Viable seeds showing red embryo coloration were counted using stereomicroscopy.

Three replicate 100-seed samples were analyzed per capsule. Statistical comparisons among pollination treatments employed chi-square tests with Bonferroni correction for multiple comparisons. Seeds lacking visible embryos were recorded separately as “empty seeds”.

### 4.9. Asymbiotic Germination Protocols

Laboratory germination trials used surface-sterilized seeds cultured on modified [66] medium at 50% standard salt concentration. Three medium formulations were tested: (1) basal MS medium; (2) MS + activated charcoal (1.0 g L^−1^); (3) MS + L-arginine (100 mg L^−1^). Each treatment included four replicate culture vessels with approximately 200 seeds per vessel.

Seed sterilization involved 15 min immersion in 2% sodium hypochlorite followed by three sterile water rinses. Cultures were maintained at 23 ± 2 °C under 16:8 h photoperiod using cool-white fluorescent illumination. Germination was scored weekly for 16 weeks, with germination defined as visible embryo swelling, chlorophyll development, and testa rupture.

Statistical analysis used one-way ANOVA with arcsin square-root transformation of percentage data. Post hoc comparisons employed Tukey’s HSD test. All analyses were performed in R version 4.3.1 [67].

## 5. Conclusions

*Govenia capitata* exhibits severe reproductive constraints driven by pollinator limitation rather than intrinsic breeding system incompatibilities. The species demonstrates complete self-compatibility with pronounced inbreeding depression, extreme habitat selectivity, and small population sizes that collectively create conservation challenges requiring targeted interventions. The successful development of asymbiotic germination protocols provides tools for ex situ conservation support, while detailed reproductive biology data guide priorities for habitat protection and restoration efforts. The species’ reproductive flexibility offers demographic insurance during conservation efforts, though genetic diversity management remains essential for long-term population viability.

These findings demonstrate the value of integrated reproductive biology and population ecology approaches for understanding conservation requirements of endemic orchids. The methods and insights developed through this study provide a framework for investigating other threatened members of Mexico’s diverse orchid flora and contribute to the broader understanding of reproductive constraints in rare plant species.

## Figures and Tables

**Table 1:** Reproductive success and seed characteristics across pollination treatments in *Govenia capitata.* Values are means ± SD. Different superscript letters indicate significant differences within columns (*p* < 0.05).

Treatment	Flowers (n)	Fruit Set (%)	Capsule Mass (g)	Capsule Width (cm)	Seed Viability (%)
Natural pollination	12	16.6	1.93 ± 0.43 ^a^	1.35 ± 0.07 ^a^	30.18 ± 8.1 ^a^
Autonomous selfing	12	0.0	—	—	—
Assisted self-pollination	12	100.0	2.63 ± 0.44 ^b^	1.12 ± 0.24 ^b^	11.30 ± 4.2 ^b^
Cross-pollination	12	100.0	3.74 ± 0.63 ^c^	1.38 ± 0.27 ^c^	32.54 ± 9.4 ^a^

**Table 2:** Floral visitor composition and behavior in *Govenia capitata*.

Order	Family	Individuals (n)	Percentage	Primary Behavior
Araneae	Thomisidae	20	60.6	Predatory ambush hunting
Araneae	Unidentified	5	15.2	Web construction
Coleoptera	Curculionidae	4	12.1	Feeding/resting
Hymenoptera	Vespidae	1	3.0	Apparent nectar seeking
Thysanoptera	Unidentified	3	9.1	Feeding on floral tissues

**Table 3:** Asymbiotic germination success in *Govenia capitata* across medium treatments and seed sources. Values are means ± SD. Different superscript letters indicate significant differences within columns (*p* < 0.05).

Medium Treatment	Cross-Pollinated (%)	Natural Pollination (%)	Self-Pollinated (%)
MS basal	22.1 ± 4.3 ^a^	19.8 ± 3.7 ^a^	14.2 ± 2.8 ^a^
MS + activated charcoal	31.4 ± 5.1 ^b^	29.7 ± 4.8 ^b^	18.3 ± 3.2 ^b^
MS + L-arginine	46.16 ± 6.8 ^c^	43.95 ± 6.2 ^c^	22.68 ± 4.1 ^c^

## Data Availability

The data presented in this study are available on request from the corresponding author due to the data are part of an ongoing study.
